# The role of adjuvant transcatheter arterial chemoembolization following repeated curative resection/ablation for hepatocellular carcinoma with early recurrence: a propensity score matching analysis

**DOI:** 10.1186/s12885-024-12396-2

**Published:** 2024-05-22

**Authors:** Kaiquan Huang, Tao Qian, Wen Chen, Mengyi Lao, Huiliang Li, Wei-Chiao Lin, Bryan Wei Chen, Xueli Bai, Shunliang Gao, Tao Ma, Tingbo Liang

**Affiliations:** 1 Department of Hepatobiliary and Pancreatic Surgery, the First Affiliated Hospital of Zhejiang University School of Medicine, 79 Qingchun Rd., Hangzhou, 310003 China; 2 Innovation Center for the Study of Pancreatic Diseases of Zhejiang Province, Hangzhou, China; 3 Zhejiang Clinical Research Center of Hepatobiliary and Pancreatic Diseases, Hangzhou, China; 4 Zhejiang Provincial Key Laboratory of Pancreatic Disease, Hangzhou, China; 5 Cancer Center, Zhejiang University, Hangzhou, China

**Keywords:** Hepatocellular carcinoma, Radiofrequency ablation, Early recurrence, Transcatheter arterial chemoembolization

## Abstract

**Background:**

The role of adjuvant transcatheter arterial chemoembolization (TACE) following repeated resection/ablation for recurrent hepatocellular carcinoma (HCC) remains uncertain. The aim of this study was to assess the effectiveness of adjuvant TACE following repeated resection or ablation in patients with early recurrent HCC.

**Methods:**

Information for patients who underwent repeated surgery or radiofrequency ablation (RFA) for early recurrent HCCs (< 2 years) at our institution from January 2017 to December 2020 were collected. Patients were divided into adjuvant TACE and observation groups according to whether they received adjuvant TACE or not. The recurrence-free survival (RFS) and overall survival (OS) were compared between the two groups before and after propensity score matching (PSM).

**Results:**

Of the 225 patients enrolled, the median time of HCC recurrence was 11 months (IQR, 6–16 months). After repeated surgery or radiofrequency ablation (RFA) for recurrent tumors, 45 patients (20%) received adjuvant TACE while the remaining 180 (80%) didn’t. There were no significant differences in RFS (*P* = 0.325) and OS (*P* = 0.072) between adjuvant TACE and observation groups before PSM. There were also no significant differences in RFS (*P* = 0.897) and OS (*P* = 0.090) between the two groups after PSM. Multivariable analysis suggested that multiple tumors, liver cirrhosis, and RFA were independent risk factors for the re-recurrence of HCC.

**Conclusion:**

Adjuvant TACE after repeated resection or ablation for early recurrent HCCs was not associated with a long-term survival benefit in this single-center cohort.

## Introduction

Primary liver cancer is the sixth most common malignancy and the third most common cause of cancer-related mortality worldwide, with approximately 830,000 deaths annually [1]. Hepatocellular carcinoma (HCC) accounts for 75% − 85% of primary liver cancers. Hepatic resection, liver transplantation, and ablation are the main curative treatments for early-stage HCC. However, recurrence of HCC after curative treatments represents a major obstacle to patient survival, with recurrence rates as high as 60% ~ 70% [2, 3] at 5 years. Early recurrence of HCC is usually defined as tumor recurrence within 2 years [4, 5], which is much common than late recurrence. Early recurrence is usually due to primary multicentric carcinogenesis or early metastatic disease, reflecting a tricky biological feature of the tumor [6, 7]. The interval between treatment and recurrence has been reported to affect patient survival after recurrence [8]. And early recurrence of HCC has been recognized as an important prognostic factor for patient survival [9, 10].

It has been shown that there is no difference in prognosis between repeated liver resection and ablation for early small recurrence of HCC [11–13]. The high rate of re-recurrence [14, 15] after repeated resection or ablation necessitate active surveillance and even adjuvant therapies following repeated treatments. However, until now, there is no consensus on whether adjuvant therapies following repeated curative resection or ablation for early recurrent HCC is necessary. Transcatheter arterial chemoembolization (TACE) is the most widely used locoregional strategy for HCC. Many studies including several randomized trials have shown that postoperative adjuvant TACE efficaciously and safely improves the recurrence-free survival (RFS) and even overall survival (OS) when compared with hepatectomy alone in patients with a high risk of recurrence [16–19]. Whether adjuvant TACE is beneficial for HCC patients who underwent repeated resection or ablation for recurrent tumors remains unclear.

This study aims to evaluate the efficacy of adjuvant TACE following repeated curative resection or ablation in patients with early recurrent HCC. Propensity score matching (PSM) was applied to control the selection bias between groups. The specific risk factors of re-recurrence after repeated curative treatments for early-recurrent HCC were also analyzed by multivariable analysis.

## Materials and methods

### Patient cohort

**Fig. 1:**
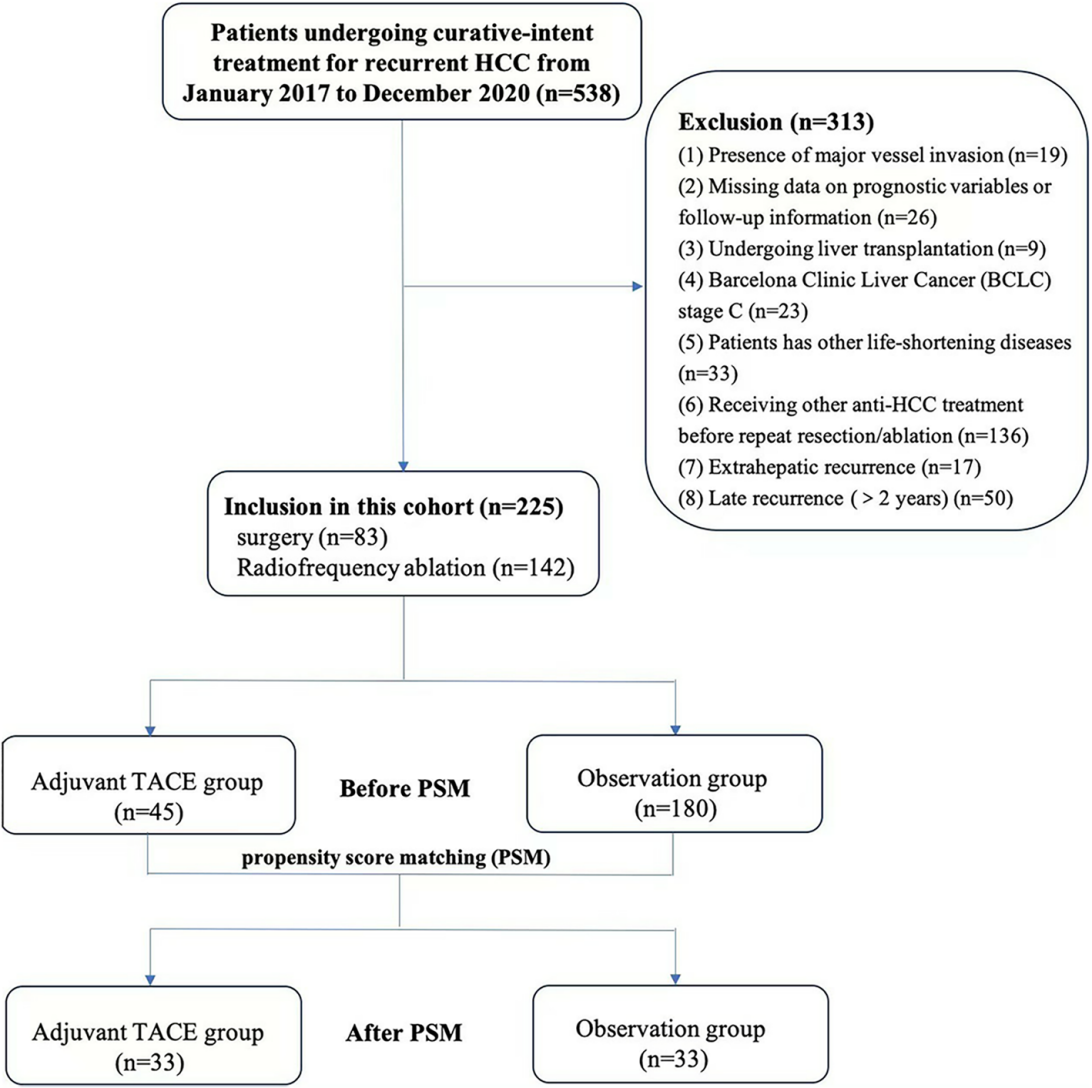
The CONSORT flow chart of this study

This study retrospectively analyzed patients who received repeated curative-intent resection or ablation for early recurrent (within 2 years postoperatively) HCC at our hospital from January 1, 2017 to December 31, 2020. Inclusion criteria were: (1) HCC was either confirmed histologically or diagnosed using noninvasive criteria according to the European Association for the Study of Liver [20]. (2) Absence of extrahepatic metastasis or major vessel invasion. (3) Patients had Eastern Cooperative Oncology Group performance status of 0 or 1. (4) Only one session of curative-intent treatment (resection or ablation) for the primary HCCs was performed before HCC recurrence that needed repeated resection or ablation. (5) A R0 resection or complete ablation were achieved for the recurrent HCC. Exclusion criteria were: (1) There were missed data on prognostic variables or follow-up information. (2) Patients who had undergone liver transplantation. (3) HCC recurrence after two years postoperatively. (4) Barcelona Clinic Liver Cancer (BCLC) stage C. (5) Severe medical system diseases (heart, lung, brain, liver, kidney, and other organ abnormalities). (6) Patients had other life-shortening malignant tumors. (7) Patients received chemotherapy, radiotherapy, and other non-surgical treatments before repeated resection/ablation. The flowchart of study design and patient enrollment is shown in Fig. 1.

### Procedures

In this study, the curative treatments of HCC included liver resection and radiofrequency ablation (RFA). Complete ablation refers to complete necrotic lesions of the local tumor tissue and no residual tumors as indicated by computed tomography (CT)/magnetic resonance imaging (MRI) one month after RFA. Curative resection was defined as a complete resection of all tumor nodules with clear microscopic resection margins. The resection methods principally involved were either anatomical or non-anatomical according to the patient’s preoperative liver reserve function assessment and tumor location. Anatomical or anatomical resections were defined by the Brisbane 2000 system [21]. Major hepatectomy was defined as the resection of three or more Couinaud segments, while minor hepatectomy involved the resection of fewer than three segments [22].

Because a global consensus or guideline on adjuvant treatments for HCC was unavailable, the clinical decision-making for choosing adjuvant therapies in our institution was usually based on the physician’s preference. For adjuvant TACE, a catheter was placed into the proper hepatic artery through the right femoral artery using the Seldinger technique, hepatic arterial angiography was performed, and 4 mg raltitrexed and 30 mg epirubicin was infused followed by 4 ~ 8 mL of the emulsion of iodized oil.

### Follow-up protocol

Liver-enhanced CT or MRI scans were performed within two weeks after the procedure to evaluate the effectiveness of hepatectomy or ablation in patients with recurrent liver cancer. Patients after curative-intent resection were followed up with CT or MRI of the liver at 1 month postoperatively and then every 3–6 months thereafter. Patients underwent liver function tests and serological tests for AFP at the same time during follow-up. The determination of tumor recurrence was based on clinical and radiographic examinations using CT or MRI, or on biopsy. All patients were followed until death or September 15, 2023. The dates of tumor recurrence, last follow-up, and death were recorded.

### Definition of outcomes

Tumor recurrence was defined as the appearance of an intra- or extra-hepatic tumor nodule by medical imaging or pathological examination. Patients with recurrent HCC were divided into 2 groups, the adjuvant TACE group and the observation group, according to whether they received adjuvant TACE or not. The primary endpoint was RFS, which was measured from the date of surgery to the dates of tumor recurrence or last follow-up. In this study, the time of recurrence was determined based on the first imaging that identified new tumors with definitive or suspicious characteristics. The report date of positive cytological or histological findings was considered as the time of recurrence for patients with confirmed recurrence through biopsy. The secondary endpoints included OS and adverse events. OS was calculated based on the time from the tumor radical treatment until death. TACE-related adverse events (AEs) were recorded according to Common Terminology Criteria for Adverse Events version 5.0 [23].

### Propensity score matching (PSM)

PSM analysis was used to reduce the differences in baseline characteristics between groups. The covariates entered in the propensity score included sex, age, etiology of liver disease, treatment modality (surgery / RFA), cirrhosis, Child–Pugh grade, BCLC staging, platelet count, HBV-DNA load, AFP levels, Gamma-glutamyl transferase levels, largest tumor size, tumor number, and anti-HBV treatment. The model offered a 1-to-1 match with a caliper value of 0.02 between the above 2 groups, as previously described.

### Statistical analysis

Statistical analysis was performed using the SPSS 26.0 statistical software packages (SPSS Inc, USA). Continuous variables were expressed as median (interquartile range, IQR). Categorical variables were reported as numbers and percentages for categorical variables. Continuous variables were tested using the Mann–Whitney U-test, and categorical variables were tested using the Fisher Test or χ 2 test. All significant factors identified in the univariate analysis were included in a binary logistic regression model to determine the independent predictors of second recurrence. RFS and OS were estimated through the Kaplan–Meier methods and were compared among the treatment groups using a log-rank test. A *P* value < 0.05 was considered statistically significant.

## Results

### Patient characteristics

The baseline characteristics of the whole cohort are shown in Table 1. A total of 538 patients have been evaluated. And among these, 313 patients have been excluded with 225 patients being included in the final analysis (190 males and 35 females). The median age was 60 (IQR, 53.5–66.5 years) years. Hepatitis B (*n* = 214, 95.1%) was the most common cause of HCC in this cohort. The median time of HCC recurrence was 11 months (IQR, 6–16 months). 83 patients (36.8%) underwent resection of recurrent tumors and the remaining 142 patients (63.1%) underwent RFA. After repeated curative-intent surgery or RFA for recurrent tumors, 45 patients (20%) received adjuvant TACE while the remaining 180 patients (80%) didn’t. Forty-two patients received one cycle of TACE and 3 patients received two or three cycles of TACE. The median follow-up period of the included patients was 49 months (IQR, 35–63.5 months). PSM was used to create 33 matched pairs of patients.

**Table 1:** Comparisons of patients’ baseline characteristics and perioperative outcomes between two group before and after PSM

		**Before PSM**			**After PSM**		
	**Total cohort** (*n* = 225)	**Adjuvant TACE group** (*n* = 45)	**Observation group** (*n* = 180)	***P*-value**	**Adjuvant TACE group** (*n* = 33)	**Observation group** (*n* = 33)	***P*-value**
**Gender** (men: women)	190(84.4%):35(15.6%)	37(82.2%):8(17.8%)	153(85%):27(15%)	0.646	28(84.8%):5(15.2%)	29(87.9%):4(12.1%)	0.720
**Age** (years)	60 (53.5 ~ 66.5)	59 (51.5 ~ 67.5)	60 (54 ~ 66)	0.568	58 (50 ~ 64.5)	61 (56 ~ 68)	0.240
**Diabetes** (yes: no)	35(15.6%):190(84.4%)	6(13.3%):39(86.7%)	29(16.1%):151(83.9%)	0.646	5(15.2%):28(84.8%)	5(15.2%):28(84.8%)	1.000
**Drinking** (yes: no)	79(35.1%):146(64.9%)	12(26.7%):33(73.3%)	67(37.2%):113(62.8%)	0.185	11(33.3%):22(66.7%)	11(33.3%):22(66.7%)	1.000
**Fatty liver** (yes: no)	9(4%):216(91%)	1(2.2%):44(97.8%)	8(4.4%):172(95.6%)	0.496	1(3%):32(97%)	2(6%):31(94%)	0.555
**HBV** (yes: no)	214(95.1%):11(4.9%)	45(100%):0(0%)	169(93.9%):11(6.1%)	0.089	33(100%):0(0%)	33(100%):0(0%)	-
**HBV-DNA** (positive: negative)	41(18.2%):184(81.8%)	14(31.1%):31(68.9%)	27(15%):153(85%)	0.012	10(30.3%):23(69.7%)	14(42.4%):19(57.6%)	0.306
**Liver cirrhosis** (yes: no)	164(72.9%):61(27.1%)	35(77.8%):10(22.2%)	129(71.7%):51(28.3%)	0.409	25(75.8%):8(24.2%)	29(87.9%):4(12.1%)	0.202
**Treatment modalities** (surgery: RFA)	83(36.9%):142(63.1%)	32(71.1%):13(28.9%)	51(28.3%):129(71.7%)	< 0.001	21(63.7%):12(36.3%)	24(72.8%):9(27.2%)	0.428
**BCLC stage** (0/A: B)	222(98.7%):3(1.3%)	44(97.8%):1(2.2%)	178(98.9%):2(1.1%)	0.561	32(97%):1(3%)	29(87.9%):4(12.1%)	0.163
**Maximum diameter** (cm)	1.6 (1.2 ~ 2.2)	1.8 (1.2 ~ 2.5)	1.6 (1.2 ~ 2)	0.118	1.7 (1.2 ~ 2.6)	1.7 (1.4 ~ 2.6)	0.681
**Tumor number** (single: multiple)	181(80.4%):44(19.6%)	30(66.7%):15(33.3%)	151(83.9%):29(16.1%)	0.009	27(81.8%):6(18.2%)	27(81.8%):6(18.2%)	1.000
**Child–Pugh class** (A: B)	208(92.4%):17(7.6%)	43(95.6%):2(4.4%)	165(91.7%):15(8.3%)	0.377	31(94%):2(6%)	30(91%):3(9%)	0.642
**γ-glutamine transferase** (U/L)	38 (24 ~ 62)	34 (24.5 ~ 54)	40 (24 ~ 63.7)	0.456	34 (24.5 ~ 52)	36 (28 ~ 71)	0.140
**Platelet** (10^9^/L)	116 (82.5 ~ 158)	118 (102 ~ 155.5)	114.5 (77.5 ~ 158)	0.350	118 (102 ~ 147)	120 (53 ~ 163.5)	0.691
**AFP** (U/L)	7.7 (2.9 ~ 46.7)	11.1 (2.5 ~ 200.3)	7.6 (3 ~ 42)	0.520	6.5 (2.4 ~ 81.7)	7.6 (3.1 ~ 35)	0.768
**Prothrombin time** (s)	12.2 (11.5 ~ 12.9)	12 (11.5 ~ 12.9)	12.2 (11.5 ~ 13)	0.565	11.8 (11.2 ~ 12.8)	12.7 (11.5 ~ 13.4)	0.124
**TBil(total bilirubin)** (μmol)	13.5 (9.2 ~ 18.6)	12 (9 ~ 17.9)	13.9 (9.2 ~ 18.9)	0.467	12.8 (10.4 ~ 20)	16.3 (11.1 ~ 20.4)	0.305
**Albumin** (g/L)	42.8 (38.7 ~ 46.1)	42 (38.2 ~ 46.2)	43 (38.7 ~ 46.1)	0.879	42.6 (38.1 ~ 46.9)	44.7 (38.7 ~ 48)	0.667
**Antiviral therapy **(yes: no)	152(67.6%):73(32.4%)	38(84.4%):7(15.6%)	114(63.3%):66(36.7%)	0.007	28(84.8%):5(15.2%)	27(81.8%):6(18.2%)	0.741
**Tumor recurrence** (yes: no)	165(73.3%):60(26.7%)	36(80%):9(20%)	129(71.7%):51(28.3%)	0.258	26(78.8%):7(21.2%)	23(69.7%):10(30.3%)	0.398
**1-year RFS rate** (%)	53.3%	51.1%	53.8%	0.738	51.5%	48.4%	0.806
**2 years RFS rate** (%)	39.1%	35.5%	40%	0.585	39.3%	36.3%	0.800
**5 years RFS rate** (%)	26.6%	20%	28.3%	0.258	21.2%	30.3%	0.398
**Survival** (yes: no)	153:72	25:20	128:52	0.045	20:13	16:17	0.323
**1-year OS rate** (%)	95.1%	91.1%	96.1%	0.164	87.8%	90.9%	0.689
**2 years OS rate** (%)	84.8%	80%	86.1%	0.306	84.8%	63.6%	0.049
**5 years OS rate** (%)	71.5%	60%	74.4%	0.055	66.6%	48.4%	0.135

*TACE* transcatheter arterial chemoembolization, *HBV* Hepatitis B Virus, *RFA* Radiofrequency ablation, *BCLC* Barcelona Clinic Liver Cancer, *AFP* Alpha-fetoprotein, *RFS* Recurrence free survival, *OS* Overall survival

### Comparisons of clinical outcomes

**Fig. 2:**
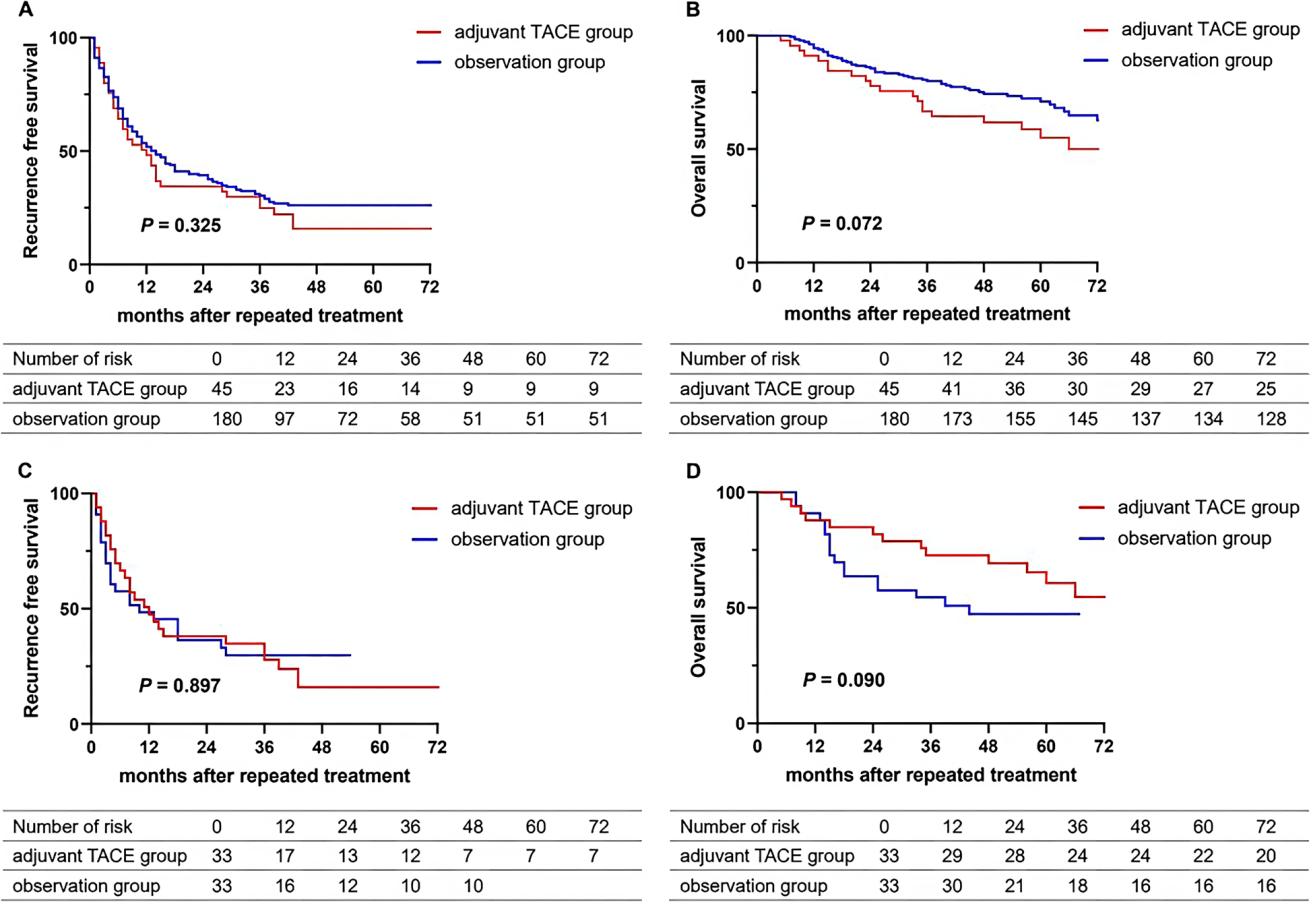
Kaplan–Meier curves for RFS and OS. **A** and **B** RFS (**A**) and OS (**B**) of patients before PSM. **C** and **D** RFS (**C**) and OS (**D**) of patients after PSM

Patients in the adjuvant TACE group more often had higher rates of viral DNA positivity (31.1% vs 15%, *P* = 0.012), antiviral therapy (84.4% vs 63.3%, *P* = 0.007), surgery (71.1% vs 28.3%, *P* < 0.001), and multiple tumors (33.3% vs 16.1%, *P* = 0.009) compared with the observation group before PSM. There were no differences in other baseline characteristics and operative variables between the two groups. (Table 1) The median RFS in the TACE group was comparable to that in the observation group (12 mo vs. 14 mo, *P* = 0.325) before PSM. And the median OS in the TACE group was also comparable to that in the observation group (unreached vs. unreached, *P* = 0.072) (Fig. 2, A and B).

All the clinical variables were balanced between patients in the TACE group and the observation group after PSM (Table 1). The median RFS in the TACE group was comparable to that in the observation group (12 mo vs. 10 mo, *P* = 0.897) after PSM. And the median OS in the TACE group was also comparable to that in the observation group (unreached vs. unreached, *P* = 0.090) (Fig. 2, C and D). Re-recurrence after repeated treatments occurred in 165 (73.3%) patients, including 36 (80%) in the TACE group and 129 (71.6%) in the observation group. A total of 54 patients had HCC re-recurrence at the resection margin, including 39 (27.4%) cases of RFA and 15 (18%) cases of surgery. Intrahepatic recurrence (146, 64.8%) occurred more frequently than distal metastasis (19, 8.4%) in the whole cohort. Moreover, the rate of locoregional recurrence in the adjuvant TACE group was comparable to that in the observation group (26.6% vs 23.3%, *P* = 0.640) in the whole cohort (Table 2).

**Table 2:** Comparisons of recurrence location between adjuvant TACE group and observation group

Location of re-recurrence	Adjuvant TACE group (*n* = 45)	Observation group (*n* = 180)	*P*-value
Local recurrence	12(26.7%)	42(23.3%)	0.640
Intrahepatic	20(44.4%)	72(40%)	0.588
Extrahepatic	1(2.2%)	5(2.8%)	0.836
Intrahepatic & Extrahepatic	3(6.7%)	10(5.6%)	0.775

*TACE* transcatheter arterial chemoembolization

The most common TACE-related AE was fever (21, 46.7%). Other AEs included abdominal pain (11, 24.4%), elevated liver enzymes (10, 22.2%), nausea/vomiting (7, 15.5%). No patients experienced an AE with severity of grade 3 or above (Table 3). No TACE-related death.

**Table 3:** Adverse Events for the adjuvant TACE group at the censor of follow-up

Adverse events to TACE	Grade of toxicity				
	**I**	**II**	**III**	**IV**	**V**
**Nausea/vomiting**	7	0	0	0	0
**Fever**	7	14	0	0	0
**Pain**	11	0	0	0	0
**Leukopenia**	0	2	0	0	0
**Liver failure**	0	0	0	0	0
**Bleeding**	0	0	0	0	0
**Liver abscess**	0	0	0	0	0
**Increase in ALT/AST**	7	3	0	0	0
**Increase in bilirubin**	5	2	0	0	0

*ALT* alanine aminotransferase, *AST* aspartate aminotransferase

### Risk factors for re-recurrence of HCC

The univariable analysis identified that liver cirrhosis, treatment modalities, tumor number, and AFP level were risk factors for re-recurrence of HCC (all *P* < 0.05). Multivariable logistic regression analysis found that liver cirrhosis (95% CI: 1.608 ~ 6.177, OR: 3.152, *P* = 0.001), multiple tumors (95% CI: 1.252–9.754, OR: 3.494, *P* = 0.017), and RFA treatment (95% CI: 1.217 ~ 4.443, OR: 2.326, *P* = 0.011) were independent risk factors for re-recurrence of HCC (Table 4).

**Table 4:** Univariate and multivariate analysis of risk factors for re-recurrence of HCC

	**Univariate analysis**			**Multivariate analysis**		
	**OR**	**95%CI**	***P* value**	**OR**	**95%CI**	***P* value**
**Gender** (men: women)	0.335	0.266 ~ 1.569	0.646			
**Age** (> 60 years vs. ≤ 60 years)	0.747	0.413 ~ 1.351	0.335			
**Diabetes** (yes: no)	1.547	0.637 ~ 3.756	0.335			
**Drinking** (yes: no)	1.007	0.542 ~ 1.870	0.983			
**Fatty liver** (yes: no)	0.717	0.174 ~ 2.962	0.646			
**HBV** (yes: no)	0.598	0.125 ~ 2.849	0.518			
**HBV-DNA** (positive: negative)	1.157	0.528 ~ 2.532	0.716			
**Liver cirrhosis** (yes: no)	2.840	1.509 ~ 5.346	0.001	3.152	1.608 ~ 6.177	0.001
**Treatment** (surgery: RFA)	1.923	1.053 ~ 3.510	0.033	2.326	1.217 ~ 4.443	0.011
**BCLC stage** (0/ A: B)	-	-	0.999			
**Maximum diameter** (> 2 vs. ≤ 2 cm)	1.265	0.661 ~ 2.418	0.478			
**Tumor number** (single: multiple)	3.405	1.273 ~ 9.103	0.015	3.494	1.252 ~ 9.754	0.017
**Child–Pugh class** (A: B)	0.863	0.291 ~ 2.560	0.790			
**γ-glutamine transferase** (> 45 vs. ≤ 45 U/L)	1.302	0.704 ~ 2.407	0.400			
**Alkaline phosphatase** (> 125 vs. ≤ 125 U/L)	1.197	0.375 ~ 3.826	0.761			
**Platelet** (> 100 vs. ≤ 100 10^9^/L)	0.833	0.443 ~ 1.564	0.569			
**AFP** (> 200 vs. ≤ 200 U/L)	3.717	1.084 ~ 12.744	0.037	1.003	1.000 ~ 1.006	0.075
**ALT** (> 40 vs. ≤ 40 U/L)	1.604	0.576 ~ 4.465	0.365			
**AST** (> 40 vs. ≤ 40 U/L)	1.532	0.594 ~ 3.953	0.378			
**Prothrombin time** (> 13.5 vs. ≤ 13.5 s)	0.587	0.282 ~ 1.223	0.155			
**TBil(total bilirubin)** (> 21 vs. ≤ 21 μmol)	1.158	0.512 ~ 2.622	0.725			
**Direct bilirubin** (> 7 vs. ≤ 7 μmol)	0.870	0.421 ~ 1.797	0.706			
**Albumin** (> 40 vs. ≤ 40 g/L)	0.671	0.348 ~ 1.292	0.233			
**Antiviral therapy** (yes: no)	0.689	0.358 ~ 1.328	0.266			
**Hospital stays** (days)	0.980	0.943 ~ 1.018	0.302			

*HBV* Hepatitis B Virus, *RFA* Radiofrequency ablation, *BCLC* Barcelona Clinic Liver Cancer, *AFP* Alpha-fetoprotein, *ALT* alanine aminotransferase, *AST* aspartate aminotransferase

## Discussion

To the best of our knowledge, this may be the first study evaluating the role of adjuvant TACE in preventing re-recurrence after repeated curative intent resection or ablation for recurrent HCC. Because of the high rate of early recurrence, many HCC patients undergo repeated surgery or ablation for recurrent tumors when they are sporadic, small in size, and are not accompanied by extrahepatic metastasis. No matter which stage the primary tumor is, early recurrence indicates more aggressive nature of the tumor and a more unfavorable prognosis. It’s reasonable to raise the question that whether the repeated surgery or ablation alone is sufficient enough in disease control. Actually, many clinicians have done the opposite. As shown in this cohort, 20% of patients received adjuvant TACE, although currently there is no evidence supporting the usage of adjuvant TACE in preventing subsequent recurrence. Considering that numerous studies have shown adjuvant TACE may reduce HCC recurrence and even prolong survival in postoperative patients who have high risk factors for recurrence [18, 24, 25], adjuvant TACE following repeated resection or ablation of HCC seems to be a potentially promising option.

In this study, patients who underwent adjuvant TACE had more chances of getting repeated surgery instead of RFA, multiple tumors, and higher HBV-DNA load. Considering the imbalance between the two groups, a PSM method was implied to reduce the selection bias. Interestingly, the RFS and OS were both comparable between groups before and after PSM. Adjuvant TACE might only bring a temporary survival advantage as the 2-year OS rate was better in patients who underwent adjuvant TACE in this study. However, given the complicated epigenetic characteristics associated with HCC, such benefit by TACE was lost over time. It’s worth mentioning that this finding should be interpreted with caution because of the relatively small sample size after PSM. Also, there was no statistical difference observed between the two groups in terms of the patterns of disease recurrence. It was proposed that postoperative TACE might improve the outcomes of HCC patients by destroying residual occult intrahepatic disease close to the tumor bed or other adjacent satellite lesions that are not identified by perioperative imaging or during surgery [18, 26, 27]. Disappointingly in the current study, although most cases of re-recurrence occurred in the liver, adjuvant TACE failed to efficiently prevent locoregional recurrence after surgery or ablation. Recently, a retrospective study including 489 patients with a low risk of recurrence (tumor size ≤ 5 cm, single nodule, no satellites, and no microvascular or macrovascular invasions) shows that the RFS in patients with adjuvant TACE is significantly lower than that in patients without, and the OS is comparable between the two group pf patients [28]. This study indicates a potential harmful effect of prophylactic TACE on the prognosis of patients with early HCC. Similarly in this cohort, although most recurrence occurred within 1 year after treatment, there were only 3 patients with a BCLC stage B disease, and the remaining 222 cases were BCLC stage 0 and A. Adjuvant TACE might be ineffective in preventing subsequent recurrence after curative resection or ablation of recurrent early-stage HCCs, while the underlying mechanism is still unknown. As mentioned above, early recurrence of HCC after curative surgery usually indicates a tricky biological feature of the tumor. Thus, a systemic approach may be an appealing alternative to adjuvant locoregional therapies in such instance. Systemic treatments of HCC mainly include tyrosine kinase inhibitors, monoclonal antibodies, and immune checkpoint inhibitors. Various drugs and their combinations have been under investigation by a variety of clinical trials in the adjuvant setting. The preliminary results of the IMbrave050 trial indicated adjuvant therapy with atezolizumab plus bevacizumab improved RFS in HCC patients following surgical resection or ablation [29]. This trial may clarify the utility of immune checkpoint inhibitors combined with monoclonal VEGF antibodies for HCC in the adjuvant setting. Long-term follow-up is needed to confirm the long-term benefits as well as the safety profile of the regimen. Unlike locoregional therapies such as TACE, combined atezolizumab and bevacizumab therapy offers a systemic response to minimal residual tumors and carcinogenesis stimuli by inhibiting tumor-related angiogenesis and tumor growth, and reversing immunosuppression thereby enhancing antitumor immune responses. These advantages may explain the superiority of combined immunotherapy and targeted therapy in the adjuvant setting.

The present study suggested that multiple tumors and liver cirrhosis were independent risk factors for re-recurrence of HCC after repeated surgery or ablation. This finding was in concordance with previous studies [30]. Interestingly, the current study also found that RFA was an independent risk factor for re-recurrence of HCC. Early recurrent tumors most likely originate from occult micro-metastasis of the primary tumor and are commonly associated with a more aggressive tumor behavior, such as large tumor size, multinodularity, poor tumor differentiation, microvascular invasion, and satellite lesions [31–33]. In contrast, late recurrent tumors often have a clonal origin that are different from the original tumors, suggesting a de novo growth pattern [34, 35]. Comparing to surgical resection, RFA only ablates a relatively limited liver volume surrounding the tumor, weakening its coverage of occult micro-metastasis, microvascular infiltration, and satellite modules around the primary tumor. Some studies report that histopathological examinations identified satellite foci in 44% of RFA-treated HCC lesions during follow-up [36, 37]. Thus, clinicians should pay more attention to early recurrent HCCs after RFA. A thorough examination including contrast enhanced CT/MRI is usually warranted to exclude other minimal recurrent nodules in the liver as well as systemic metastases.

Postembolization fever occurred in nearly half of patients. TACE related AEs should be paid more attention especially in the adjuvant setting. Some studies have even shown that patients with TACE may have a worse prognosis due to the toxic effects of TACE procedure [38, 39]. Considering the low efficiency of adjuvant TACE in the current study, adjuvant TACE may be better not recommended to patients with early recurrent HCC who undergo repeated curative resection/ablation.

The present study has several limitations. Firstly, the single-center retrospective nature of this study and a relatively small sample size brought significant selection bias, although a PSM method was applied. Secondly, because data were limiting, we can’t evaluate and compare the efficacy of other adjuvant treatments with TACE. Finally, due to the relatively short period of follow-up, this study did not achieve the median OS in both groups.

In summary, this study first evaluated the utility of adjuvant TACE following repeated curative resection or ablation for patients with early recurrent HCC. Disappointingly, we found adjuvant TACE neither improved the RFS nor OS in patients who underwent repeated surgical resection or ablation for recurrent HCC, comparing to observation. Further prospective studies are needed to validate this finding.

## Conclusions

Adjuvant TACE for early recurrent HCCs was not associated with a long-term survival benefit in this single-center cohort.

## Data Availability

All data generated or analysed during this study are included in this published article.

## Abbreviations

TACE: Transcatheter arterial chemoembolization
HCC: Hepatocellular carcinoma
RFA: Radiofrequency ablation
RFS: Recurrence-free survival
OS: Overall survival
PSM: Propensity score matching
IQR: Interquartile range
BCLC: Barcelona Clinic Liver Cancer
CT: Computed tomography
MRI: Magnetic resonance imaging
AE: Adverse event
HBV: Hepatitis B virus
DNA: Deoxyribonucleic acid
AFP: Alpha fetoprotein
CI: Credibility interval
